# Exploring the diagnostic value of eosinophil-to-lymphocyte ratio to differentiate Kawasaki disease from other febrile diseases based on clinical prediction model

**DOI:** 10.1038/s41598-023-30463-9

**Published:** 2023-02-28

**Authors:** Xin Guo, Jinwen Liao, Xue Fan, Mingguo Xu

**Affiliations:** 1 Department of Pediatrics, Longgang District Maternity & Child Healthcare Hospital of Shenzhen City, Shenzhen, 518172 China; 2 Science and Education Section, Shenzhen Longgang District Third People’s Hospital, Shenzhen, 518115 China

**Keywords:** Diagnostic markers, Predictive markers, Cardiovascular diseases, Infectious diseases

## Abstract

Kawasaki disease (KD) is a febrile disease that affects children under 5 years of age and leads to serious cardiovascular complications such as coronary artery disease. The development of markers that can predict early is important to reduce the under- and misdiagnosis of KD. The aim of this research was to develop a diagnostic predictive model to differentiate Kawasaki disease (KD) from other febrile diseases using eosinophil-to-lymphocyte ratio (ELR) and other biomarkers. We recruited a total of 190 children with KD and 1604 children with other febrile diseases. We retrospectively collected clinical information from the children, which included laboratory data on the day of admission, such as white blood cells (WBC), hemoglobin (HGB), calcitoninogen (PCT), hypersensitive c-reactive protein (CRP), snake prognostic nutritional index (PNI), peripheral blood neutrophil–lymphocyte ratio (NLR), platelet-lymphocyte ratio (PLR), and ELR. We performed analyses using univariate analysis, multivariate logistic regression, and column line plots, and evaluated the diagnostic parameters of the predictive models. ELR was significantly increased in patients with KD. After multivariate logistic regression, WBC, HGB, CRP, NLR, ELR and PNI were finally included as indicators for constructing the prediction model. The ROC curve analysis suggested that the C-index of the diagnostic prediction model was 0.921. The calibration curve showed good diagnostic performance of the columnar graph model. The cut-off value of ELR alone for KD was 0.04, the area under the ROC curve was 0.809. Kids with KD show highly expressive level of ELR compared to children with febrile disease, which can be used to diagnose KD, and column line graphs constructed together with other indicators can help pediatricians to identify KD more effectively from febrile children.

## Introduction

Kawasaki disease (KD) is a febrile disease that affects children under 5 years of age^1^. The core pathological feature is systemic vasculitis, which is common in medium and small-sized vessels. The severe complication is coronary artery lesion (CAL), including coronary aneurysm, coronary stenosis, coronary thrombosis and sudden cardiac death, etc^2^. In recent years, with the increased incidence of KD, especially incomplete KD and KD unresponsive to IVIG therapy, KD has evolved as a major cause of acquired heart disease in children and adolescentsn^3^.

The etiology and pathogenesis of KD are not fully understood, but may be related to a combination of genetics, post-infection stress, and immune response^4^. It has been suggested that invasion by specific pathogens and disturbances in immune regulation may result in KD^5^. Therefore, it has been speculated that inflammatory parameters associated with exogenous post-infection stress have the potential to predict KD and its prognosis^6^. Recent research have shown that blood cell parameters neutrophil-to-lymphocyte ratio (NLR) and platelet-to-lymphocyte ratio (PLR) are associated with IVIG resistance in KD^7^.

Eosinophils in the blood are an important component of the immune system and have a role in regulating over-activation of immune function and promoting inflammatory responses^8^. Chemokines and cytokines all stimulate the accumulation of eosinophils, while eosinophil activation can also secrete cytokines to further promote the inflammatory cascade response^9^. Study shows eosinophils can effectively differentiate KD from other febrile diseases^10^. Meanwhile, eosinophils were also found to be associated with IVIG resistance in KD^11^. The eosinophil-to-lymphocyte ratio (ELR) is a well-recognized indicator of inflammation^12^. Studies have shown that ELR is significantly elevated in patients with allergic rhinitis, suggesting an association with allergic reactions and inflammation^13^. ELR has also been found to have better diagnostic accuracy than eosinophils or lymphocytes alone in predicting coronary slow flow phenomena^14^. From the above, it is clear that ELR is closely associated with neocoronary pneumonia, allergic rhinitis, and abnormal coronary blood flow. Recent manuscripts have also confirmed that eosinophils can be used to differentiate Kawasaki disease from other febrile diseases, but with low diagnostic performance. As mentioned earlier, ELR is more accurate than eosinophils in the diagnosis of coronary slow flow abnormalities, however, the value of ELR in differentiating KD from other febrile diseases has not been reported yet.

The diagnosis of KD depends on clinical phenotype, and there is no controversy in confirming the diagnosis of complete KD, but the confirmation of incomplete KD or suspected cases is often more difficult, which can easily lead to misdiagnosis and missed diagnosis. Timely application of IVIG treatment in the acute phase of KD can reduce the incidence of CAL^15^. Therefore, the purpose of this study was to investigate the diagnostic value of ELR for KD in an attempt to provide a basis for decision making for early intervention in KD and prevention of serious complications of KD.

## Results

### Clinical features and univariate analysis

To demonstrate the clinical and laboratory characteristics of the two populations, descriptive statistical analyses and one-way analysis of variance were performed on the parameters of the two populations. The results of the normal distribution test suggested that age, WBC, HGB, PCT, CRP, NLR, PLR, ELR and PNI did not conform to a normal distribution. A total of 1794 children were enrolled in this study, of whom 1089 (61%) were male and 705 (39%) were female. 121 (64%) boys and 69 (36%) girls were in the KD group, with a median age of 1.00 (1.00–2.00) years-old in the KD group. There were 968 (60%) boys and 636 (40%) girls in the febrile control group with a median age of 1.00 (1.00–2.00) years-old. We next performed univariate analysis of all variables and found that comparing gender and age in two groups suggested no statistical significance (p > 0.05), while comparing WBC, HGB, PCT, CRP, NLR, PLR, ELR and PNI in two groups suggested statistical significance (Table 1).

**Table 1:** Phenotype of study subjects between the KD and the febrile controls.

	Overall, N = 17,941	Febrile controls, N = 16,041	KD, N = 1901	*p*-value
Age(years-old)	1.00 (1.00–2.00)	1.00 (1.00–2.00)	1.00 (1.00–2.00)	0.074
Gender, N (%)				0.373
Female	705 (39%)	636 (40%)	69 (36%)	
Male	1089 (61%)	968 (60%)	121 (64%)	
WBC (× 10^9^/L)	10.1 (7.3–14.3)	9.6 (7.0–13.6)	15.0 (10.9–18.0)	< 0.001
HGB (g/L)	117 (110–123)	117 (111–124)	110 (103–116)	< 0.001
PCT (ng/mL)	0.13 (0.05–0.48)	0.11 (0.05–0.41)	0.43 (0.15–1.72)	< 0.001
CRP (mg/L)	13 (3–37)	11 (1–28)	63 (45–97)	< 0.001
NLR	1.17 (0.61–2.26)	1.06 (0.58–2.02)	2.56 (1.51–4.43)	< 0.001
PLR	79 (56–115)	77 (54–109)	110 (79–159)	< 0.001
ELR	0.02 (0.01–0.06)	0.02 (0.01–0.05)	0.09 (0.04–0.16)	< 0.001
PNI	61 (55–71)	62 (56–71)	55 (47–61)	< 0.001

### Multivariate logistic regression analysis

The indicators with statistical differences in the univariate analysis, such as WBC, HGB, PCT, CRP, NLR, PLR, ELR and PNI, were included in the logistics regression equation analysis to find the independent risk factors for KD, and the nomogram were drawn according to the statistically significant results in logistics regression analysis. Thus, the results of the logistics regression analysis showed that WBC, HGB, CRP, NLR, ELR and PNI were the independent risk factors for KD compared to other febrile diseases (Table 2).

**Table 2:** Multivariate logistic regression analysis between the KD and the febrile controls.

	*p*-value	*OR*	95% confidence interval of *OR*	
			Lower	Upper
WBC (× 10^9^/L)	< 0.001	1.165	1.113	1.219
HGB (g/L)	< 0.001	0.962	0.943	0.982
PCT (ng/mL)	0.639	1.015	0.953	1.082
CRP (mg/L)	< 0.001	1.015	1.01	1.021
NLR	0.001	0.835	0.748	0.931
PLR	0.749	1.000	0.997	1.002
ELR	< 0.001	931,505.992	54,334.399	15,969,688.254
PNI	< 0.001	0.901	0.874	0.929
Constant	< 0.001	245.284	14.349	4192.945

### Evaluation model for predicting KD

**Figure 1:**
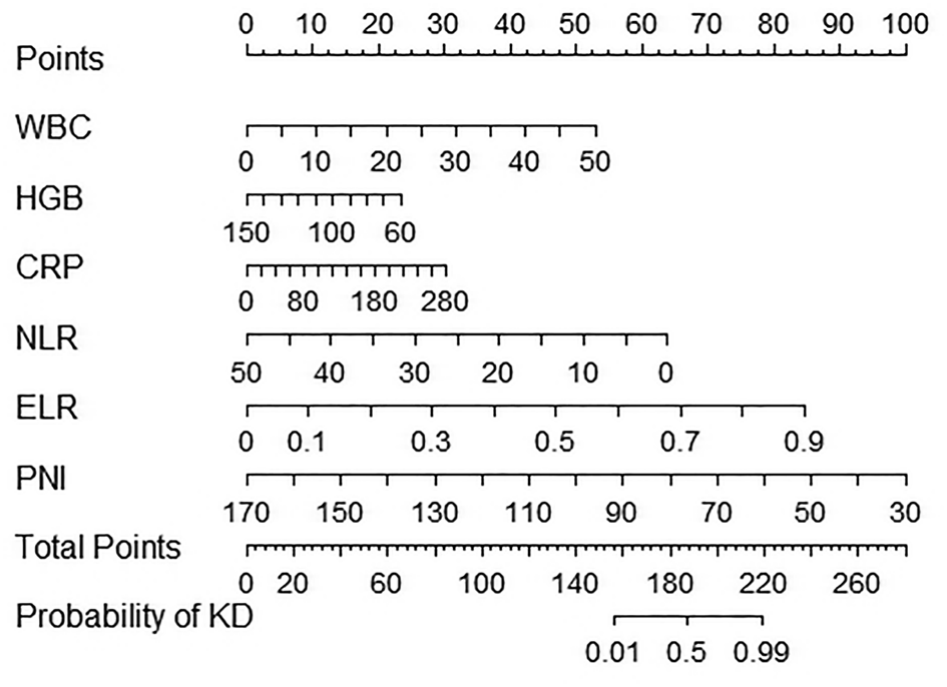
Nomogram plot of KD diagnostic prediction model. WBC (× 10^9^/L); HGB (g/L); CRP(mg/L).

We plotted the nomogram using the rms package in the R software. The results of the multivariate logistic regression were used to plot the nomogram (Fig. 1). The corresponding line chart of the web page can be viewed at https://guoxinlinar.shinyapps.io/KDvsfever/. The risk of occurrence of KD corresponding to the total score in the nomogram is shown in Table 3. As shown in Fig. 1, the "Points" at the top are the points corresponding to each index below, for example, if the "ELR" of a febrile child is 0.5, the "ELR" in the For example, if the ELR of a febrile child is 0.5, the corresponding score of "ELR" in the model is about 45. The "Total Points" in the graph are the total points obtained by summing the scores of all indicators, and the total points correspond to the probability of KD in the "Probability of KD" in the graph. The web version we have developed is the same as this chart, but it is easier to use.

**Table 3:** The total score in the column line graph corresponds to the risk of KD.

Total points	Risk of KD
140	0.001
156	0.01
167	0.05
172	0.1
178	0.2
182	0.3
185	0.4
188	0.5
190	0.6
193	0.7
197	0.8
203	0.9
208	0.95
219	0.99
235	0.999

### Performance of the nomogram

**Figure 2:**
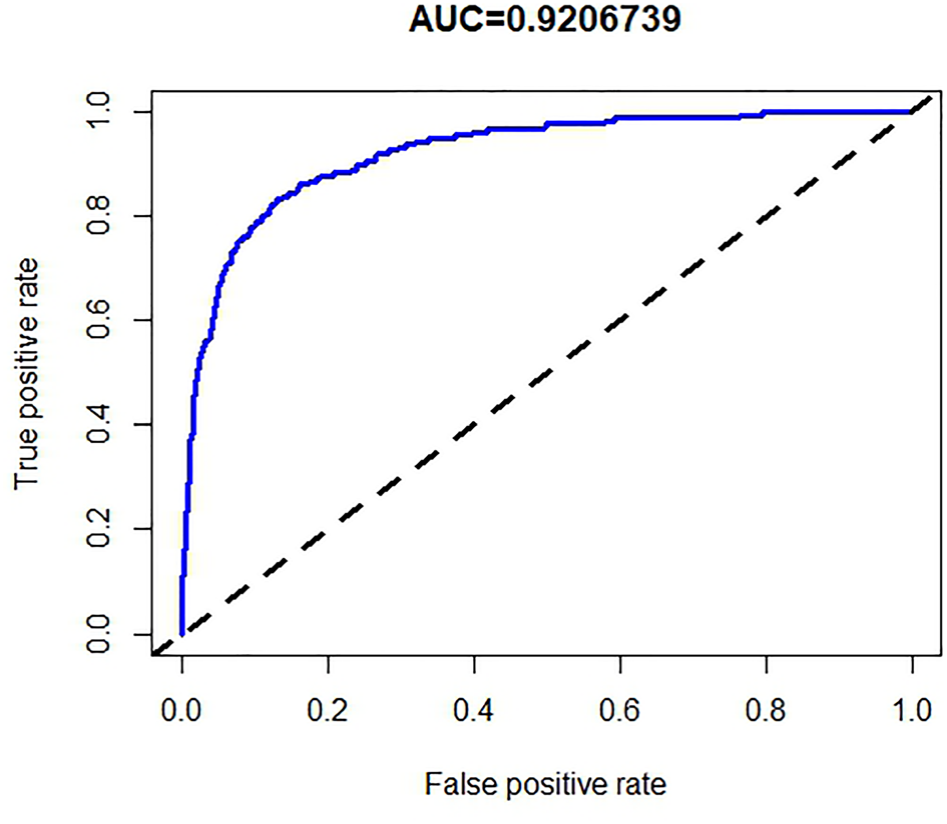
ROC curve plot corresponds to the nomogram.

**Figure 3:**
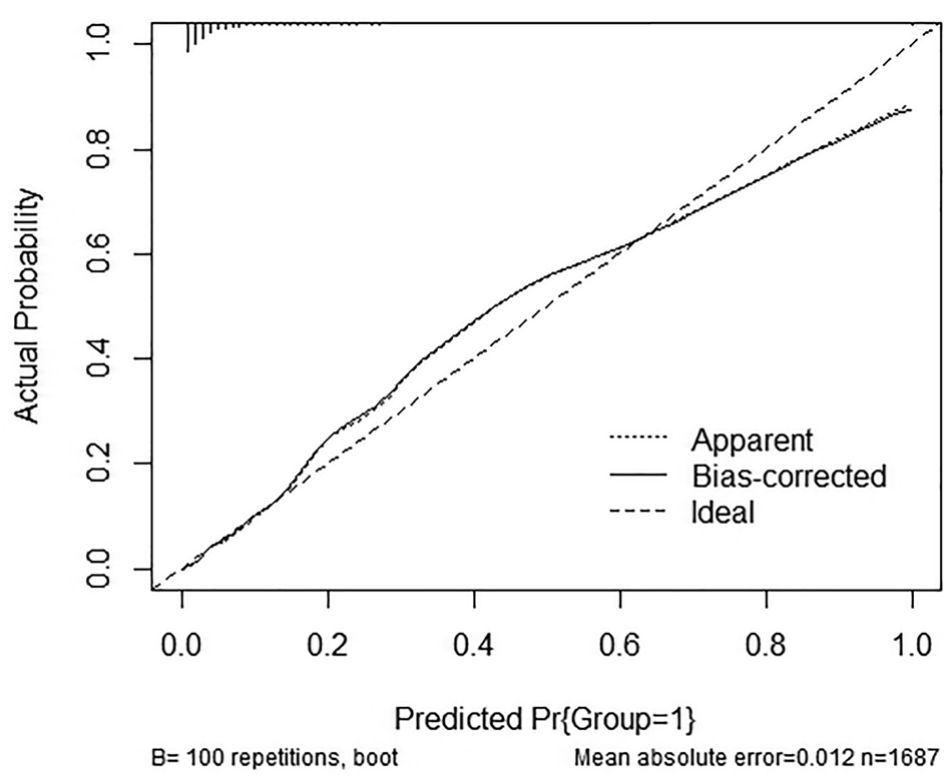
The calibration curves of the nomogram.

We plotted the ROC curves of the prediction model using the ROCR package in the R software. The ROC curve to examine the diagnostic performance of the column line graph showed an area under the curve of 0.921(95% CL: 0.900–0.942), suggesting good diagnostic performance (Fig. 2). The C-index of the column line plot was also 0.921 (95% CL: 0.900–0.942) and was confirmed by bootstrapping validation to be 0.918, which indicates that the model has good discriminatory power. A calibration curve was used to internally validate the diagnostic performance of the column line graph^16^. The results of the calibration curve showed that the predicted probability of the column line diagram for KD basically matched well with the actual probability, which suggested a good correspondence between the predicted and actual results (Fig. 3).

### ROC curve analysis of KD diagnosed by ELR alone

**Figure 4:**
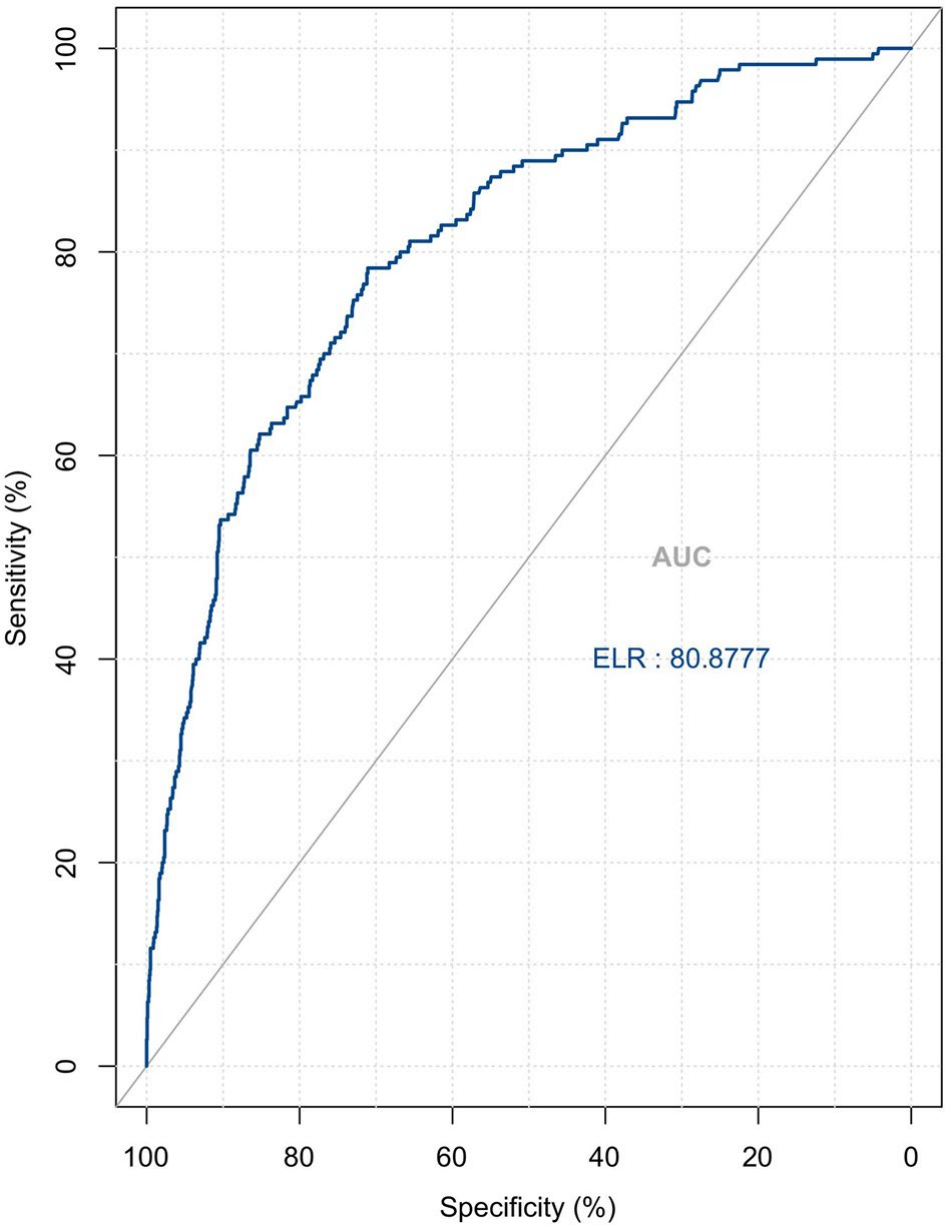
ROC curve for KD diagnosed by ELR. AUC = 0.809.

To test the diagnostic value of ELR alone in discriminating KD from other febrile diseases, we performed a separate ROC curve analysis of ELR levels in both groups. The area under the ROC curve for ELR was 0.809 (Fig. 4). the cutoff value for ELR was 0.04 (Table 4).

**Table 4:** The cutoff of the ELR.

	Cutoff	Sensitivity	Specificity	Youden index
ELR	0.04	0.784	0.711	0.495

## Discussion

In this report, we reviewed clinical information from 190 patients with KD and 1604 children with other febrile illnesses, analyzed the ability of ELR to discriminate between children with KD and other febrile illnesses, and focused on the construction of a new highly accurate prediction model. We successfully constructed a clinical diagnostic model based on logistic regression with six factors including WBC, HGB, PCT, CRP, NLR, PLR, ELR and PNI. The results of ROC curve, calibration curve and bootstrapping validation showed that the model has good accuracy and stability.

Mr. Tomisaku Kawasaki, a Japanese pediatrician, came into contact with his first child with Kawasaki disease in 1961 and first reported his collection of 50 Kawasaki disease patients in English in 1974^17^. His research revealed that a subset of children with unexplained fever would develop similar symptoms of skin, mucous membrane and lymph node changes, and would also cause serious cardiovascular complications such as coronary artery thrombosis. Kawasaki disease has become one of the leading causes of acquired heart disease in children as more and more cases are reported^3^. The early diagnosis of KD is still highly dependent on the specific clinical signs and symptoms of KD as in the past, and the signs and symptoms of KD in clinical practice are sometimes less typical or incompletely present at the early stage of the disease, which sets a barrier to the confirmation of KD diagnosis. During the three years of the COVID-19 epidemic, the diagnosis of KD has been given a new challenge because of the increasing reports of multisystem inflammatory syndrome in children (MIS-C), a complication of COVID-19 infection in children with signs and symptoms highly similar to those of KD^18^. Unlike other COVID-19 patients, children with MIS-C have their own unique clinical phenotype and organ damage pattern^19^. Cardiovascular complications and inflammatory cytokine storms are common features of MIS-C and KD^20^. The high incidence of cardiac complications, elevated inflammatory markers, elevated markers of myocardial damage, and thrombocytopenia and lymphopenia have been noted in studies as diagnostic tools to differentiate MIS-C from KD^21^. The time frame for our selected study population was January 2018 to June 2022 in Shenzhen, with the COVID-19 epidemic continuing from the initial December 2019 until today. A Japanese cohort study showed a significant reduction in the incidence of children with KD during the COVID-19 epidemic compared to the pre-COVID-19 epidemic^22^. The epidemiological trend of COVID-19 in Shenzhen was characterized by a rapid initial growth of the epidemic, followed by a short period of peak incidence, and then a rapid reduction in incidence, all due to the rapid development of non-pharmaceutical interventions and measures by the Shenzhen government to effectively control community and intra-family transmission^23^. Control measures in South China during the COVID-19 epidemic resulted in a significant reduction in respiratory pathogen infections, which may be related to the reduced incidence of KD in Shenzhen^24^. Of course active COVID-19 vaccination also has an indirect effect on the reduction of respiratory infections and the reduction of Kawasaki disease. Few cases of MIS-C have been reported in Shenzhen, and incomplete KD and atypical KD are still the main sources of misdiagnosis and underdiagnosis in the diagnosis of KD.

Confirmation of the diagnosis of KD remains a considerable challenge for pediatricians, as the clinical presentation of KD is sometimes incomplete and delayed, coupled with the interference of COVID-19 infection and Multisystem Inflammatory Syndrome, making it difficult to distinguish KD from other febrile illnesses in children based on clinical presentation alone, and there are currently no specific laboratory tests for KD to assist in confirming the diagnosis. A recent study reported on the epidemiology and independent risk factors for delayed diagnosis of KD, which found that delayed diagnosis occurs in one in ten KD cases initially, but has gradually decreased to 4.6% over time. Children older than 1 year, with atypical clinical presentation and incomplete KD were associated with late diagnosis, while delayed diagnosis was strongly associated with the occurrence of CAL^25^. Pediatricians need accurate diagnosis of KD and timely treatment of KD in order to reduce the incidence of CAL. Therefore, clinical prediction models are an important tool to assist pediatricians in more accurately identifying KD from other febrile illnesses.

WBC expression has been shown to be significantly increased in the acute phase of KD and should be considered as one of the alternative predictors^26^. Although WBC alone is not very specific for the diagnosis of KD, it can improve the identification of KD when combined with other indicators^27^. WBC can also be used to predict the risk of CAL and the odds of non-response to IVIG therapy in KD patients^28^. Therefore, although the diagnostic ability of WBC for KD is limited, it is still widely used in actual clinical work, and its diagnosis of KD still has some clinical significance.

Transient anemia has been shown to occur in children with the acute phase of KD, which may be associated with iron deficiency induced by the inflammatory factor iron-regulator^29^. HGB can be combined with other indicators to differentiate KD from other febrile children^30^. A decrease in HGB after IVIG treatment is associated with IVIG resistance^31^. The ability of HGB alone to diagnose KD remains limited, but HGB is still a predictor worth considering.

CRP is the classic clinical indicator of inflammation and is significantly elevated during the acute phase of KD^32^. CRP can likewise be combined with other indicators to distinguish KD from other febrile children to compensate for its own lack of diagnostic specificity^33^. CRP can be combined with other inflammatory indicators to predict the risk of IVIG resistance in children with KD^34^. As an extension of its predictive value, the ratio of CRP to albumin can be used to predict coronary artery disease and IVIG resistance in children with KD^35^. These demonstrate the role of CRP in predicting KD and its complications.

High NLR expression is strongly associated with the severity of KD^36^. NLR together with other indicators are relatively effective in distinguishing KD from other febrile children^37^. NLR combined with other factors can predict KD coronary lesions^38^. NLR was also an important predictor of IVIG resistance in children with KD^7^.

PNI is used to assess the immune and nutritional status of patients and has been reported as a predictive marker of surgical prognosis in various types of cancer^39^. Studies have demonstrated that PNI can be used to predict KD from febrile patients^27^. Additional studies have identified PNI as a novel independent predictor of IVIG-resistant KD^7^. PNI also has diagnostic value for KD shock syndrome and cardiovascular complications^40^.

Studies have shown increased expression of eosinophils in children with KD, suggesting that eosinophils are associated with the development of Kawasaki disease^41^. Eosinophils can be used as one of the indicators to differentiate between children with KD and hyperthermia^42^. In addition, studies have found a strong correlation between eosinophils and coronary lesions in KD^43^. ELR as an indicator of inflammation has not been discussed for its clinical significance in KD. In our study, we found for the first time that ELR expression was significantly increased in KD compared to other febrile children. When the ELR is greater than 0.04, there is reason to highly suspect the possibility of KD. The diagnostic accuracy of KD can then be increased by combining it with other inflammatory indicators.

This is a single-center retrospective study with a relatively small number of cases, and the prediction model needs to be further validated in a multicenter and larger sample cohort study.

Our study showed that inflammatory markers such as WBC, HGB, CRP, NLR, ELR, and PNI have good predictive ability for KD. This report is the first to cite ELR as a predictor to discriminate KD from other febrile diseases. This paper clarifies the importance of ELR for KD and provides a direction for further exploration on the pathogenesis of KD.

## Methods

### Study design and ethics statement

The trial design was developed in accordance with the STROBE statement of observational study guidelines^44^. The type of research was categorized as a case-control study. This study was conducted according to the guidelines of the Declaration of Helsinki and approved by the ethics committee of Shenzhen Longgang District Maternity & Child Healthcare Hospital (protocol code IRB No. LGFYYXLLL-2022-025 in 2022.09.29). The Medical Ethics Committee of Longgang District Maternity & Child Healthcare Hospital of Shenzhen City has approved this retrospective study to waive the subject's informed consent.

### Study setting and study participants

Children with KD hospitalized at Shenzhen Longgang District Maternity & Child Healthcare Hospital from January 2018 to June 2022 were included in this study. Children with fever of other etiologies during the same period were used as controls^27^. 36 KD patients and 9140 febrile control patients were excluded because of autoimmune disease, genetic metabolic disease, unexplained fever, or incomplete data. Finally 190 KD patients and 1604 febrile control patients were included in the study.

### Data sources and variables

The research data came from information collected from clinical medical record systems by two researchers within the team. There were no differences in gender and age between the two groups of children, suggesting comparability between the groups (Table 1). Clinical and laboratory indices were collected and compared between the two groups of children. The diagnosis of KD and incomplete KD was based on the KD diagnostic criteria established by the American Heart Association (AHA) in 2017^15^. The age and gender were obtained from the first page of the medical case system and were filled in by the child and his relatives. The routine blood and biochemical results are the actual data uploaded by the hospital's laboratory department, and the test samples were taken on the day the child was admitted to the hospital and before the IVIG was used. Information on gender, age, weight, clinical presentation, and laboratory results were collected from enrolled children, including laboratory results such as white blood cells (WBC), hemoglobin (HGB), calcitoninogen (PCT), hypersensitive C-reactive protein (CRP), NLR, PLR, ELR, Onodera's prognostic nutritional index (PNI). PNI is composed of a serum albumin value (ALB) and a lymphocyte count with the formula PNI = ALB (g/L) + 5 × lymphocyte count (10^9^/L). The formula for calculating ELR is: ELR = Eosinophil count (10^9^/L)/lymphocyte count (10^9^/L).

### Bias controls and study size

Patients with fever in KD and non-KD were included in the study strictly according to the diagnostic criteria. The study was retrospective, and two subject members masked patient name and hospitalization number information while deriving information from the medical record system to reduce selection bias in the study. Both data collectors were experienced pediatricians who were familiar with the inclusion and exclusion criteria and workflow of the study, laboratory data were measured uniformly by a laboratory physician according to the criteria, and the laboratory physician did not know in advance that the subject being tested would be included as a study subject, which reduced information bias. Routine blood, blood biochemistry and KD are influenced by age and gender, so the included control patients in this study will match the age distribution and gender distribution of Kawasaki disease patients to reduce confounding bias caused by confounding factors. We estimated the sample size required for the study using PASS software and found that 8 KD patient and 8 control patient were expected to be required. The sample size was calculated by setting a category one error α = 0.05 and a category two error β = 0.2. The results of the calculations indicated that the actual sample size included in the study far exceeded the estimated sample size.

### Statistical analysis

We used R-4.2.1 software and the rms and ROCR statistical package for statistical analysis and visualization. Firstly, To demonstrate the clinical and laboratory characteristics of the two populations, descriptive statistical analyses and one-way analysis of variance were performed on the parameters of the two populations. That is, we will test for normal distribution for the measures involved in the two groups. The measurement data conforming to the normal distribution are statistically described by means and standard deviations and analyzed for differences by independent samples t-test; the non-normally distributed measurement data are statistically described by median and interquartile range (IQR) and analyzed for differences by Mann–Whitney U rank sum test. Count data were statistically described by frequencies and percentages, and differences were analyzed by chi-square test. p-values < 0.05 suggested that the differences were statistically significant. Next, parameters with statistically significant analysis of variance were screened for inclusion in the logistic regression equation for regression analysis. statistically significant parameters in the logistic regression equation were included in the final logistic regression prediction model. roc curves, calibration curves, and bootstrapping validation to calculate C-index were used to validate the prediction model for accuracy and stability^45^. The nomogram, calibration curve, and ROC curve are plotted using R software.

## Data Availability

The raw data sets involved in the current study are not publicly available due to hospital confidentiality agreement requirements, but are available from X.G. and M.X. upon reasonable request.

## References

[1] Harrison M, Scalici P (2022). Clinical guideline highlights for the hospitalist: Management of Kawasaki disease. J. Hosp. Med..

[2] Fukazawa R (2020). JCS/JSCS 2020 guideline on diagnosis and management of cardiovascular sequelae in Kawasaki disease. Circ. J..

[3] Yamashita M (2017). Difference in risk factors for subtypes of acute cardiac lesions resulting from Kawasaki disease. Pediatr. Cardiol..

[4] Nagata S (2019). Causes of Kawasaki disease-from past to present. Front. Pediatr..

[5] Nakamura A, Ikeda K, Hamaoka K (2019). Aetiological significance of infectious stimuli in Kawasaki disease. Front. Pediatr..

[6] Parthasarathy P, Agarwal A, Chawla K, Tofighi T, Mondal T (2015). Upcoming biomarkers for the diagnosis of Kawasaki disease: A review. Clin. Biochem..

[7] Li G, Xu X, Chen P, Zeng R, Liu B (2021). Prognostic value of pretreatment prognostic nutritional index in intravenous immunoglobulin-resistant Kawasaki disease. Heart. Vessels..

[8] Jacobsen E (2021). Eosinophil knockout humans: Uncovering the role of eosinophils through eosinophil-directed biological therapies. Annu. Rev. Immunol..

[9] Nakagome K, Nagata M (2020). Possible mechanisms of eosinophil accumulation in eosinophilic pneumonia. Biomolecules.

[10] Liu X (2020). A nomogram model identifies eosinophilic frequencies to powerfully discriminate Kawasaki disease from febrile infections. Front. Pediatr..

[11] Kuo H (2007). The relationship of eosinophilia to intravenous immunoglobulin treatment failure in Kawasaki disease. Pediatr. Allergy Immunol..

[12] Damar Çakırca T, Torun A, Çakırca G, Portakal R (2021). Role of NLR, PLR, ELR and CLR in differentiating COVID-19 patients with and without pneumonia. Int. J. Clin. Pract..

[13] Yenigun A, Sezen S, Calim O, Ozturan O (2016). Evaluation of the eosinophil-to-lymphocyte ratio in pediatric patients with allergic rhinitis. Am. J. Rhinol. Allergy..

[14] Tosu A (2022). Association of eosinophil-to-lymphocyte ratio with coronary slow-flow phenomenon in patients undergoing coronary angiography. Arch. Med. Sci. Atheroscler. Dis..

[15] McCrindle B (2017). Diagnosis, treatment, and long-term management of Kawasaki disease: A scientific statement for health professionals from the American Heart Association. Circulation.

[16] Alba A (2017). Discrimination and calibration of clinical prediction models: Users' guides to the medical literature. JAMA.

[17] Kawasaki T, Kosaki F, Okawa S, Shigematsu I, Yanagawa H (1974). A new infantile acute febrile mucocutaneous lymph node syndrome (MLNS) prevailing in Japan. Pediatrics.

[18] Feldstein L (2020). Multisystem inflammatory syndrome in U.S. children and adolescents. N. Engl. J. Med..

[19] Feldstein L (2021). Characteristics and outcomes of US children and adolescents with multisystem inflammatory syndrome in children (MIS-C) compared with severe acute COVID-19. JAMA.

[20] DeBiasi R (2021). Multisystem inflammatory syndrome of children: Subphenotypes, risk factors, biomarkers, cytokine profiles, and viral sequencing. J. Pediatr..

[21] Godfred-Cato S (2022). Distinguishing multisystem inflammatory syndrome in children from COVID-19, Kawasaki disease and toxic shock syndrome. Pediatr. Infect. Dis. J..

[22] Ae, R. *et al. *Incidence of Kawasaki disease before and after the COVID-19 pandemic in Japan: Results of the 26th Nationwide Survey, 2019 to 2020. *JAMA. Pediatr.*10.1001/jamapediatrics.2022.3756 (2022). 10.1001/jamapediatrics.2022.3756 PMC9577881 36251290

[23] Xu S (2021). Analysis of the comprehensive non-pharmaceutical interventions and measures in containing the COVID-19 epidemic in Shenzhen: A retrospective study. BMJ Open.

[24] Wang H (2022). Lockdown measures during the COVID-19 pandemic strongly impacted the circulation of respiratory pathogens in Southern China. Sci. Rep..

[25] Mat Bah M, Alias E, Sapian M, Abdullah N (2022). Delayed diagnosis of Kawasaki disease in Malaysia: Who is at risk and what is the outcome. Pediatr. Int..

[26] Chaudhary H (2019). Biomarkers for Kawasaki disease: Clinical utility and the challenges ahead. Front. Pediatr..

[27] Huang Y (2021). Complement 3 and the prognostic nutritional index distinguish Kawasaki disease from other fever illness with a nomogram. Children.

[28] Muto T (2019). White blood cell and neutrophil counts and response to intravenous immunoglobulin in Kawasaki disease. Glob. Pediatr. Health..

[29] Huang Y, Kuo H (2017). Anemia in Kawasaki disease: Hepcidin as a potential biomarker. Int. J. Mol. Sci..

[30] Yang Y (2022). Combination of hemoglobin-for-age Z-score and plasma hepcidin identified as a novel predictor for Kawasaki disease. Children.

[31] Atsumi Y (2020). Decreased hemoglobin after initial treatment is associated with treatment resistance in Kawasaki disease in Kobayashi risk stratification. World J. Pediatr..

[32] Liu X (2020). A novel nomogram model for differentiating Kawasaki disease from sepsis. Sci. Rep..

[33] Zandstra J (2020). Biomarkers for the discrimination of acute Kawasaki disease from infections in childhood. Front. Pediatr..

[34] Liu C, Wu J (2022). Value of blood inflammatory markers for predicting intravenous immunoglobulin resistance in Kawasaki disease: A systematic review and meta-analysis. Front. Pediatr..

[35] Tsai C, Yu H, Tang K, Huang Y, Kuo H (2020). C-Reactive protein to albumin ratio for predicting coronary artery lesions and intravenous immunoglobulin resistance in Kawasaki disease. Front. Pediatr..

[36] Cho H (2017). High neutrophil: Lymphocyte ratio is associated with refractory Kawasaki disease. Pediatr. Int..

[37] Yan JH (2020). Clinical characteristics for differentiating febrile children with suspected Kawasaki disease diagnosis. Front. Pediatr..

[38] Chang L (2020). Neutrophil-to-lymphocyte ratio and scoring system for predicting coronary artery lesions of Kawasaki disease. BMC Pediatr..

[39] Nogueiro J (2022). The impact of the prognostic nutritional index (PNI) in gastric cancer. Langenbecks Arch. Surg..

[40] Liu X (2022). Predictive role of sampling-time specific prognostic nutritional index cut-off values for intravenous immunoglobulin resistance and cardiovascular complications in Kawasaki disease. Int. Immunopharmacol..

[41] Kwak J, Lee J, Ha K (2019). Significance of differential characteristics in infantile Kawasaki disease. Korean Circ. J..

[42] Tsai C (2021). A novel score system of blood tests for differentiating Kawasaki disease from febrile children. PLoS ONE.

[43] Chen Y, Guo M, Chang L, Kuo H (2022). The impact of onset age on eosinophils in Kawasaki disease. Biomedicines..

[44] Cuschieri S (2019). The STROBE guidelines. Saudi J. Anaesth..

[45] Wang H (2018). Predicting medication nonadherence risk in a Chinese inflammatory rheumatic disease population: Development and assessment of a new predictive nomogram. Patient Prefer. Adherence.

